# Highly Efficient Adsorption of Pb(II) by Functionalized Humic Acid: Molecular Experiment and Theoretical Calculation

**DOI:** 10.3390/ma16237290

**Published:** 2023-11-23

**Authors:** Qi Xu, Yan Yan, Yazhou Jiao, Jinxiong Wu, Xiuling Yan, Xintai Su

**Affiliations:** 1 University and College Key Lab of Natural Product Chemistry and Application in Xinjiang, School of Chemistry and Chemical Engineering, Yili Normal University, Yining 835000, China; xq18356276765@163.com (Q.X.); 13136887951@163.com (Y.Y.); yazhoujiao0213@163.com (Y.J.); 2 Key Laboratory of Pollution Control and Ecosystem Restoration in Industry Clusters (Ministry of Education), School of Environment and Energy, South China University of Technology, Guangzhou 510006, China; suxintai827@163.com

**Keywords:** HA, adsorption, lead, complexation

## Abstract

Environmental pollution has been widely considered by researchers, especially the heavy metals damage to the human and ecological environment is irreversible. Adsorption is an important method to remove heavy metal ions from the environment. In this paper, humic acid (HA) was functionalized by the improved Hummers method, and its adsorption capacity for Pb(II) was studied. The results of scanning electron microscope (SEM), X-ray diffraction (XRD), Roman, and Brunauer-Emmett-Teller (BET) showed that the thickness of irregular particles decreases to a layered structure during the transformation process. In addition, X-ray photoelectron spectroscopic (XPS) and Fourier transform infrared spectra (FT-IR) spectra showed that the surface of oxidized-biochar (OBC) was rich in reactive oxygen species, which was conducive to the formation of coordination bonds with Pb(II). Further adsorption experiments showed that it was a spontaneous monolayer chemisorption. The results of the DFT calculation showed that -COOH had the lowest adsorption energy for Pb(II), and it was easier to form stable chemical bonds than -OH, -C=O, and -C-O-C-. Because those oxygen-containing functional groups not only can promote electrostatic attraction but also are more favorable for forming a covalent bond with Pb(II). This study had guiding significance for the deep modification and application of weathered coal as a heavy metal ion adsorbent or cation exchanger.

## 1. Introduction

In recent decades, a large amount of wastewater, gas, and residue has been discharged into the natural water environment, causing serious pollution to water bodies [1]. Heavy metal pollution is the focus of people’s attention, especially because of its toxicity at low concentrations and ease of accumulation in organisms [2]. Pb(II) is particularly noted for its irreparable harmful effects on the human body [3]. Low levels of lead could cause kidney damage and nervous system diseases, while its high levels can cause high blood pressure, joint pain, and gene mutation in humans [4,5,6,7,8]. Therefore, it is imperative to exploit sustainable and green solutions to eliminate or reduce the pollution and harm of lead ions.

Various ways have been used to treat wastewater containing lead ions, including complexation, precipitation, microbiological, membrane technology, and adsorption. [9,10,11,12,13] However, most of those ways have the disadvantages of high cost, poor selectivity, and secondary pollution. Therefore, the adsorption technique has been proverbially used [14]. Carbon-containing adsorbents are widely used due to their special pore structure, large specific surface area, and good stability [15,16]. A large number of documents have reported the application of carbon materials as adsorbents to remove heavy metal ions, such as carbon nanosheets, [17] carbon nanotubes [18], nanostructured carbon materials, and so on [19,20,21]. However, some drawbacks are their limited practical application, for example, poor dispersion and expensive or complicated fabrication procedures [22]. Therefore, it is worthwhile to develop a simple adsorbent with abundant active functional groups.

It is assumed that oxygen-containing functional groups can be used as adsorption sites for heavy metal ions because these functional groups remove heavy metal ions through forming coordination bonds, so the preparation of a carbon material rich in active adsorption sites is the focus of this work. As we all know, HA is a natural macromolecule, environmentally friendly, non-toxic, and green biomass material [23]. The structure of the HA macromolecule consists of aromatic and alicyclic rings [24] and functional, groups such as carboxy, hydroxyl, carbonyl, or methoxy groups, attached to the ring [25]. Because of its rich active functional groups, it has been widely used in the environment [26,27]. The content of carbon in HA is more than 65%, which is an ideal precursor for preparing carbon materials [28]. However, it is still a big challenge to judge what kind of oxygen-containing functional groups are most conducive to adsorption and how to effectively control the aggregation of such functional groups.

In this work, we applied carbon-rich HA as a raw material through the modified Hummers method synthesized OBC. We hypothesized that catalytic oxidation technology could produce highly efficient and easily separate and inherited original and added more reactive functional groups rather than relying on surface area and porous channels. Similar literature has not been reported so far. The aqueous solution of Pb(II) was compared with OBC and HA as adsorbents, and adsorption properties were compared. In addition, the interaction mechanism between Pb(II) was studied through SEM, FT-IR, and XPS.

## 2. Experiment

### 2.1. Materials and Chemicals

HA came from Double Dragons Humic Acid Co., Ltd., Ürümqi, China. Potassium permanganate (KMnO_4_) and sodium hydroxide (NaOH) were obtained from Shanghai Aladdin Biochemical Technology Co., Ltd., Shanghai, China. Sodium nitrate (NaNO_3_), 30% hydrogen peroxide (H_2_O_2_), sulfuric acid (H_2_SO_4_), hydrochloric acid (HCl), and lead nitrate (Pb(NO_3_)_2_) were received from Shanghai Macklin Biochemical Co., Ltd., Shanghai, China. Experimental deionized water was prepared by Smart-Q15 (Hitech, Shanghai, China).

### 2.2. Preparation of OBC

All chemicals were of pure analytical grade. Firstly, 48 mL H_2_SO_4_, 1 g NaNO_3_, and 6 g HA were added into a three flask, and then 3 g KMnO_4_ was slowly added, undergoing oxidative stripping at different temperatures. Finally, 10 mL H_2_O_2_ was added to remove excess MnO_4_^−^ [29,30,31]. Rinse the product with deionized water until the pH is close to 7.0, then obtain OBC. HA-400 was obtained by calcining HA at 400 °C in an N_2_ atmosphere for 2 h.

### 2.3. Adsorption Experiment

The effects of pH, pollutant concentration, and adsorption time on the adsorption were studied. The adsorption model, kinetics, and thermodynamics were also estimated. The selectivity and regeneration of the adsorbent were also studied.

An amount of OBC and Pb(II) aqueous solution was added to the conical flask for Pb(II) adsorption tests. The suspension solution was then thoroughly vibrated at 200 rpm using a mechanical shaker (THZ-100B, China Trading Co., Ltd., Shanghai, China) at 25 °C. Then, the liquid was filtered by a 0.22 μm filter membrane, and the concentration of Pb(II) was detected by ICP-OES (Avio 200 and Optima 8000, PerkinElmer, Waltham, MA, USA). Study on the effect of pH on adsorption in the case of adsorbent mass 5.0 mg and pollutant concentration 50 mg·L^−1^. The initial concentration of Pb(II) ranges from 10 to 100 mg·L^−1^ for adsorption isotherm experiments. The kinetic experiments were implemented with contact time from 2 to 120 min.

The adsorption efficiency of Pb(II) is calculated as follows: [32]

(1) $$R=\frac{\left(C_{0}-C_{e}\right)}{C_{0}}$$

(2) $$q_{t}=\frac{(C_{0}-C_{t})V}{m}$$

where *C*_0_ and *C_e_* (mg·L^−1^) are the initial concentration and equilibrium concentration of lead, and *V* (mL) is the of the pollutant solution, *m* (mg) is the quality of the adsorbent.

#### 2.3.1. Effect of pH on Adsorption

The effects of pH on adsorption in the range of 2.0–7.0 were studied to investigate the optimal pH value.

#### 2.3.2. Adsorption Isotherm

Under the condition of pH = 6.0, the concentration of contaminant was in the range of 20–100 mg·L^−1^, and the adsorption performance is studied by ionic strength. The adsorption isotherms were studied by using the Langmuir isotherm (Equation (3)) and the Freundlich isotherm (Equation (4)) [33].

(3) $$q_{e}=\frac{q_{\max}\cdot K_{L}\cdot C_{e}}{\left(1+K_{L}\cdot C_{e}\right)}$$

(4) $$q_{e}=K_{F}\cdot C_{e}^{1/n_{F}}$$

where *K_L_* is Langmuir’s constant, *C_e_* (mg·L^−1^) is the concentration of Pb(II) at the equilibrium point, and *q*_max_ (mg·g^−1^) is the maximum adsorption amount. *K_F_* is the Freundlich constant, and *n* is adsorption intensity.

#### 2.3.3. Dubinin-Radushkevich

Under the condition of pH = 6.0, the concentration of contaminant was in the range of 20–100 mg·L^−1^, and the adsorption performance is studied by ionic strength. The adsorption isotherms were studied by using the Dubinin-Radushkevich isotherm (Equations (5)–(7)) [34].

(5) $$\ln q_{e}=\ln Q_{d}-\beta \varepsilon^{2}$$

(6) $$\varepsilon =RT\ln\left(1+\frac{1}{\mathrm{Ce}}\right)$$

(7) $$E=\frac{1}{\sqrt{2\beta }}$$

where *β* (mol^2^·kJ^−2^) is a constant related to adsorption energy, *ε* (kJ·mol^−1^) is Polanyi adsorption energy, and *E* (kJ·mol^−1^) is Polanyi adsorption energy.

#### 2.3.4. Adsorption Kinetics

The pseudo-first-order (Equation (8)) and pseudo-second-order (Equation (9)) kinetics models [6] were used to evaluate the kinetic parameters of Pb(II) adsorption on the OBC: [35]

(8) $$\ln(q_{e}-q_{t})=\ln q_{e}-k_{1}t$$

(9) $$\frac{1}{q_{t}}=\frac{1}{k_{2}q_{e}^{2}}+\frac{t}{q_{t}}$$

where *q_t_* is the adsorption capacity at time *t* (s) and *q_e_* (mg·g^−1^) the equilibrium adsorption capacity. *k*_1_ (min^−1^) and *k*_2_ (g·(mg·min)^−1^) are the rate constants of the two models, respectively.

#### 2.3.5. Adsorption Thermodynamics

The effect of temperature (298.15 K, 308.15 K, 318.15 K) on the adsorption of lead was studied. The thermodynamic parameters were evaluated by the following equations: [36]

(10) $$\ln k_{d}=\frac{C_{A}}{C_{e}}$$

(11) $$\Delta G=-RT\ln k_{d}$$

(12) $$\ln k_{d}=\frac{\Delta S}{R}-\frac{\Delta H}{RT}$$

where *R* (8.314 J·(mol·K)^−1^) is the gas constant, *C_A_* is the reduced concentration of ions in a solution at equilibrium, *T* (K) is temperature, and *k_d_* is the thermodynamic equilibrium constant. Δ*G* (J·(mol·K)^−1^) is the entropy change, Δ*H* (kJ·mol^−1^) is the enthalpy change, and Δ*S* (kJ·mol^−1^) is the Gibbs free energy change in a given process.

#### 2.3.6. Desorption and Regeneration

The cycle performance of the adsorbent is that the solution is desorbed at pH = 1.0 for 6 h, washed to neutral, freeze-dried to obtain the production, adsorbed again, and recycled 5 times.

### 2.4. Theoretical Calculation Model and Parameter

In this experiment, the adsorption process of OBC was simulated by Materialstudio 2019 software. Coronene was used as the main structure of the material, and hydroxyl, carboxyl, carbonyl, and ether bonds were connected. In the calculation process, geometry optimization and calculating the adsorption energy used generalized-gradient approximation (GGA), Perdew-Wang 91 (PW91) module in Dmol^3^, energy = 5.0 e^−5^ eV·atom^−1^, Max. Force = 0.1 eV·Å^−1^, Max. Stress = 0.2 GPa, Max. Displacement = 0.05 Å.

The calculation formula for adsorption energy is as follows:

(13) ΔE=E(A…B)−E(A)−E(B)

ΔE is the adsorption energy (Kcal·mol^−1^), *E*(*A* … *B*) is the energy of the optimized adsorption structure (Ha), *E*(*A*) is the energy of the optimized OBC (Ha), and *E*(*B*) is the energy of lead ion after optimization (Ha).

### 2.5. Characterization

The N_2_ adsorption-desorption isotherms were utilized by Brunauer-Emmett-Teller (ASAP 2020, Micromeritics, Norcross, GA, USA). The XRD of the materials was checked by an X-ray diffractometer (D8 ADVANCED, Bruker, Mannheim, Germany). A Fourier transform infrared spectrometer (Thermo, iS50, Waltham, MA, USA) was conducted to obtain FT-IR to analyze the interaction between materials. A Raman microscope (DXR2xi, Thermo) was used to acquire the Raman spectra of HA and OBC. The morphological characteristic of stacked nanosheets was examined using a Regulus 8100 scanning electron microscope (SEM) (Hitachi-hightech, Tokyo, Japan) and conducted on a JEOL-2011F transmission electron microscopy (TEM) (JEOL, Tokyo, Japan) conducted at 200 kV. XPS was analyzed using an Escalab 250Xi instrument (Thermo Fisher Scientific, Waltham, MA, USA) with an A1 Kα X-ray source. The zeta potential of the material was determined using an Omni zeta potential analyzer (Brookhaven, Holtsville, NY, USA).

## 3. Results and Discussion

### 3.1. Characterization of OBC

**Figure 1:**
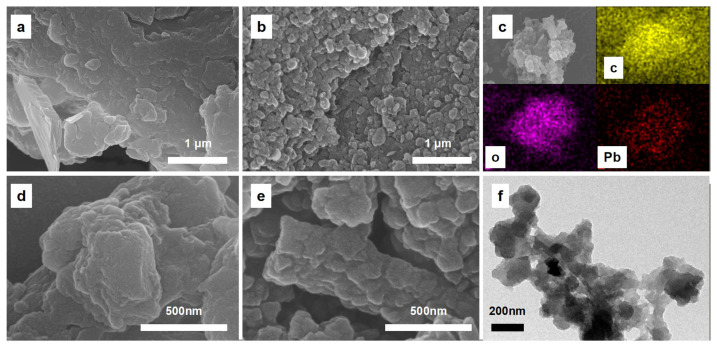
(**a**,**d**) are the SEM of HA (1 μm and 500 nm), (**b**,**e**) are the SEM of OBC (1 μm and 500 nm), (**c**) is the EDS of OBC after adsorption, and (**f**) is the TEM of OBC.

The SEM and TEM images of the materials are shown in Figure 1. The irregular particles in the HA were largely converted into a stacked structure of the OBC, and the size of the nanosheet is about 300 nm, which corresponds to the TEM image of the OBC (Figure 1f). This structure increases the specific surface area of the material and helps to bind to contaminants.

**Figure 2:**
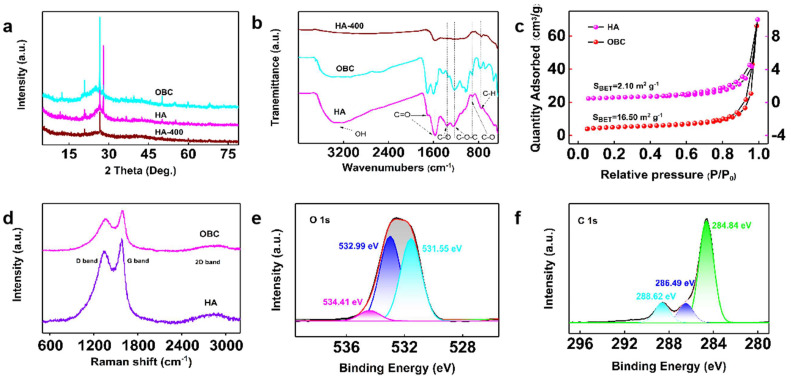
(**a**,**b**) are XRD and FTIR spectrogram of the three materials, respectively, (**c**,**d**) are BET and Roman spectra of HA and OBC, (**e**,**f**) are O 1s and C 1s energy spectra of OBC.

Figure 2a shows the X-ray diffraction peaks of HA and OBC. The diffraction peak at 2θ = 26.6° (002) was obviously weakened, and the distance between the OBC layers was 0.334 nm, calculated by Bragg law. Accordingly, OBC had enhanced crystallinity, and the layered structure of the crystal lattice was ordered. The nitrogen adsorption-desorption curves for OBC, HA-400, HA, and the adsorbent BJH pore size distribution are shown in Figure 2c and Table 1. The nitrogen adsorption-desorption isotherm of OBC belongs to the type IV isotherm, indicating that OBC has a typical mesoporous structure. Figure 2d and Table 2 show the Raman spectra and concrete value of HA and OBC. It was well known that I_D_/I_G_ can measure the disorder degree of carbon materials and the average size of sp2 planar structure, which can be used to judge the order degree of materials. By comparison, the shape of all the peaks became wider, and I_D_/I_G_ decreased, which indicated that the defect structure and the irregular carbon at the edge of the sheet were repaired. The 2D band peaks slightly increased and blue-shifted, which indicated that the degree of sheet stacking increased.

Figure 2b shows the FT-IR spectra of HA, HA-400, and OBC. The broad band shifted 3348 cm^−1^ near 3200 cm^−1^, which was caused by the edge of hydroxy stretching vibration. [37] A band of 1601 cm^−1^ may not only be related to the sp2 hybridization of C=C aromatic but can also be associated with OH bending vibration of adsorption of water molecules [38]. What’s more, C=O stretching vibration peaks of carboxyl and carbonyl groups at 1704 cm^−1^ were enhanced significantly, suggesting that more oxygen-containing functional groups were introduced into the OBC. The peak at 1573 cm^−1^ was red-shifted, indicating that oxidants had a strong interaction with graphene-like sheets [39]; 1361 cm^−1^ and 1230 cm^−1^ were caused by OH stretching vibration on the benzene ring and C-O-C vibration. The peak at 775 cm^−1^ was observed by aromatic ring C-H plane bending vibration. As a result, after Hummers method oxidation, the OBC has a more ordered aromatic carbon structure and more rich oxygen-containing functional groups.

The structure of the material was further studied by the XPS spectrum. As Figure 2e,f shown, the OBC is composed of C and a high content of O element. In the case of C 1s, the high-resolution spectra are divided into four peaks at about 288.62, 286.49, and 284.84 eV, which belong to the bonding structures of carbonyl, C-OH, C-O-C, C-H, and C=C [40]. In the C 1s spectrum, the proportion of carbon-carbon bonds is the maximum. The results show that the molecular structure of the benzene ring is more orderly, and more active functional groups are added during the conversion process of the material. Except for a small part of H_2_O, the great mass are effective functional groups. Therefore, the physical properties of HA remain stable after functionalization, but the chemical structure of HA has changed significantly; its molecular structure is mainly an aromatic ring, and the oxygen-containing functional groups on the ring increase obviously. And the structural optimization is shown in picture in Section 3.3.

### 3.2. Adsorption of Pb(II) on OBC

**Figure 3:**
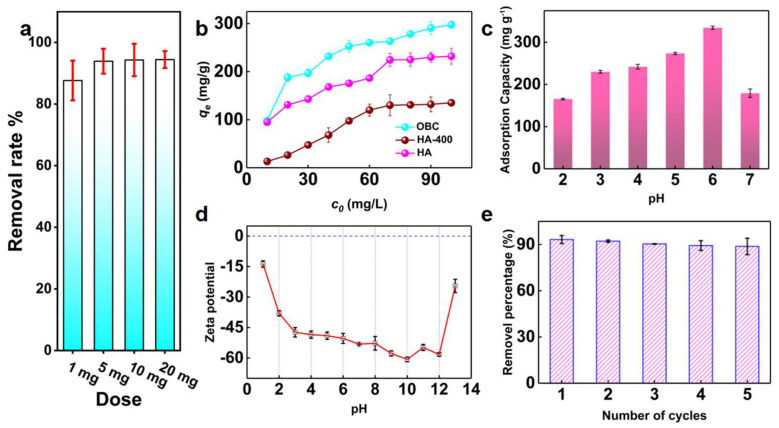
(**a**) is the influence of the amount of adsorbent on the adsorption performance, (**b**) is the adsorption performance comparison diagram of HA, OBC, and HA-400, (**c**) is a comparison of the adsorption capacity of OBC for lead ion at different pH values, (**d**) is the Zeta potential plot of OBC, (**e**) is a plot of regeneration performance of the OBC.

Firstly, as Figure 3a shows, the influence of the amount of adsorbent on the adsorption performance was studied. We added 1.0, 5.0, 10.0, and 15.0 mg OBC, respectively, under the solution concentration, was 20 mg·L^−1^, pH was 6.0, and the solution volume was 50 mL, and the results showed that the removal rate was as high as 93.9% at the dose of 5.0 mg. For economic consideration, 5.0 mg was selected as the best adsorbent dose.

We compared the adsorption properties of OBC when the dosage of adsorbent was 5.0 mg, and the initial concentration ranged from 10 to 100 mg·L^−1^ (Figure 3b). The adsorption capacity of activated carbon increased with the increase in lead concentration. When the concentration reaches a certain value, the adsorption process reaches the equilibrium of saturated adsorption capacity. The results illustrated that the maximum adsorption capacity of OBC could reach 297.72 mg·g^−1^, which was 1.28 times that of HA and 2.2 times of HA-400, which originated from the addition of rich oxygen-containing functional groups during the formation of the material. The complexation ability with Pb(II) in wastewater is greatly improved, and the adsorption capacity of HA-400 is the weakest because its effective functional group is destroyed in the roasting process. Although the specific surface area of HA increased slightly after calcination (Table 1), the adsorption performance of HA-400 decreased significantly (Figure 2b), for the effective functional groups of HA decreased sharply due to high temperature, and it could not form chemical bonds with lead ions. Therefore, it can be inferred that the adsorption process is dominated by chemical action and has no connection with physical adsorption. As shown in Table 3, we compared the Pb(II) adsorption performance of biochar with different methods to improve the active functional group and found that the biochar in this work is a kind of adsorbent with great potential.

In the process of adsorption, the pH of the solution is very important to the adsorption performance. The existing forms of lead ions in the solution were different at different pH values. With the increase of pH value, lead ions in the solution were gradually transformed from ionic form into Pb(OH)^+^, Pb(OH)_2_, Pb(OH)^+^·H_2_O and Pb(OH)_2_·H_2_O, which was not conducive to the adsorption [45]. Meanwhile, with the increase of solution pH, the deprotonation degree of the material surface increases, and the surface potential gradually decreases, as shown in Figure 3d; when pH = 10.0, the surface potential is the lowest. Figure 3c can elucidate that when the pH of the solution is 6.0, the adsorption performance is the best because the existence form of lead and the electrostatic attraction of negatively charged materials is the strongest under this condition.

#### 3.2.1. Adsorption Isotherm

**Figure 4:**
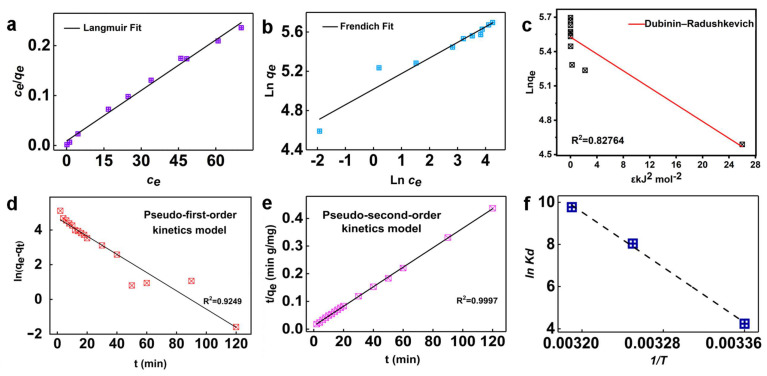
(**a**,**b**) are Freundlich and Langmuir adsorption plots of the OBC, (**c**) is Dubinin-Radushkevich linear fit plot of OBC, (**d**) is a pseudo-first-order kinetic linear fit plot of OBC, (**e**) is a pseudo-second-order kinetic linear fit plot of the OBC, (**f**) is the thermodynamic parameter diagram of OBC.

Langmuir and Freundlich models were used to fit the adsorption isotherms of lead on OBC at 298 K, corresponding to homogeneous and heterogeneous adsorption, respectively. The fitting parameters and isotherms are shown in Figure 4a,b and Table 4. The Langmuir isotherm (R^2^ = 0.999) is more suitable for this experiment by comparing R^2^ and average percent error (APE). The equilibrium adsorption capacity of OBC was 297.72 mg·g^−1^ in accordance with theoretical adsorption capacity in Langmuir models (295.86 mg·g^−1^). K_L_ is an important parameter in Langmuir, which is related to the adsorption energy and is used to predict the behavior of the adsorption path. This constant has four kinds of possibilities: 0 < K_L_ < 1, the adsorption process is feasible, and K_L_ > 1, the adsorption process is not feasible; K_L_ = 1, the adsorption process is linear, and K_L_ = 0, indicating that the adsorption process is irreversible. And When 0.1 < 1/n < 0.5 in Freundlich models, adsorption is easy. While 1/n > 2, adsorption is difficult. Although the correlation of Freundlich models is poor, 0.1 < 1/n < 0.5, which is well-retained with Langmuir isotherm. Therefore, the adsorption should be understood as the homogeneous adsorption of single molecules [46,47,48].

#### 3.2.2. Dubinin-Radushkevich Isotherm Model

Dubinin-Radushkevich isotherm model assumes that the surface of the fixed adsorbent is not uniformly hooked or that it is carried out under constant adsorption activation free energy conditions. The linear fitting diagram of the model and related parameters are shown in Figure 4c and Table 5. The R^2^ value calculated by this model is relatively low, indicating that the adsorption process is not consistent with the model [49].

#### 3.2.3. Adsorption Kinetics

In order to further study the adsorption kinetics of lead on OBC, the pseudo-first-order and pseudo-second-order kinetic models were used to fit the experimental data of lead adsorption. The kinetic isotherm parameters are shown in Figure 4d,e and Table 6. The correlation coefficient (R^2^ = 0.9997) was calculated and showed that the pseudo-second-order model can better describe the adsorption process. The pseudo-second-order kinetic model is considered to represent the linear relationship between the adsorption rate and the concentration of two reactants (including adsorbent and adsorbent), so the amount of adsorbent is an important factor. Therefore, the process is mainly controlled by chemical adsorption, including the electrostatic attraction between OBC and lead ions.

#### 3.2.4. Adsorption Thermodynamics

The effect of temperature on the adsorption process provides a basis for the energy conversion of the adsorption process. As shown in Figure 4f and Table 7, the amount of adsorption increases with increasing temperature. This may be due to the increase in temperature, which is conducive to the entry of lead ions into the sample. The thermodynamic parameters were estimated by Van’t Hoff analysis. And ΔG < 0, the adsorption process is endothermic [50,51].

#### 3.2.5. Regeneration

In order to investigate the possibility of regeneration of OBC, several desorption experiments were carried out, as shown in Figure 3e. The results showed that OBC could be regenerated. Even after five recirculations, there was no significant decrease.

### 3.3. Adsorption Mechanisms

**Figure 5:**
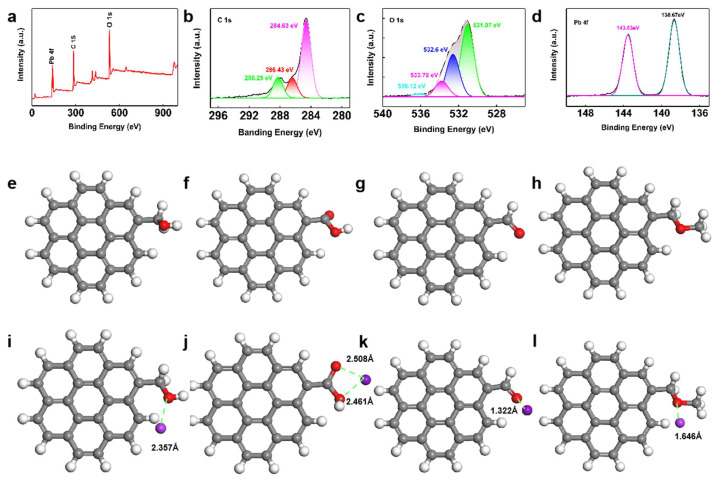
(**a**–**d**) are the XPS spectra of lead adsorbed by OBC and the energy spectra of C 1s O 1s and Pb 4f, respectively, (**e**–**h**) is the structure optimization diagram of different functional groups of OBC, and (**i**–**l**) is the structure optimization diagram of lead adsorbed by OBC.

In the adsorption process, the surface charge of the material determines the selectivity of pollutants. The surface potential of the material at different pH was studied with a Zeta potentiometer (Figure 3d). The results show that the surface Zeta potential of the material was less than zero at any pH, which provided the possibility of loading lead ions. The XPS spectrum of OBC adsorbed Pb(II) (Figure 5a–d) showed that the area of C 1s spectrum had no obvious change except that the position of binding energy shifted slightly, which indicated that the main carbon structure of OBC had not changed and the oxygen-containing functional groups had not been reduced. However, the area of O 1s satellite peak at 531.07 eV had no change. The reason was that the polar covalent bond is formed between -OH and Pb, and the area of the satellite peaks at 532.6 and 533.78 eV was reduced because the oxygen functional groups such as -C-O and -C = O polymerize with Pb(II) ions to form a covalent bond. At the same time, Pb 4f 5/2 and 4f 7/2 have two obvious peaks at 143.63 and 138.67 eV, respectively, with an energy gap of 4.96 eV, which is due to the formation of the Pb-O bond. The FTIR spectra at 3351 cm^−1^ and 1704 cm^−1^ were found to be related to the coordination of -OH and C-O (carboxyl and carbonyl) groups on OBC, respectively; 1246 cm^−1^ was the combination of in-plane bending vibration and Pb(II) [52,53]. The peak at 688 cm^−1^ is Pb-O. The results were consistent with the above analysis.

Further calculated by density functional theory (DFT) and using the generalized gradient approximation to determine the most stable structure and the lowest energy of each substance, the OBC with different functional groups after adsorption was shown in Figure 5e–1 after structural optimization, and it was found that E (…-COOH) < E (…-OH) < E (…-C=O) < E (…-O-) (Table 8). Therefore, the excellent adsorption performance of OBC was due to the fact that the OBC has more oxygen-containing functional groups, wherein the carboxyl functional group is most favorable for adsorption because the carboxyl functional group can reduce the surface potential of the OBC, enhance electrostatic attraction and is favorable for forming a covalent bond with Pb(II) ions [54,55,56].

Therefore, the adsorption mechanism may, through electrostatic gravity, attract lead ions that are positively charged to the surface of the material, and then its effective adsorption sites and oxygen-containing functional groups remove lead ions by complexing. -COOH has the lowest adsorption energy and embodies the main role, detailed in the following equation:

…-COOH+ Pb^2+^→[…-COOPb]^+^+H_2_O

…-COOH+Pb(OH)^+^→[…-COOPb]^+^+H_2_O

…-COOH+ Pb(OH)^+^·H_2_O→[…-COOPb]^+^+2H_2_O

2…-COOH+ Pb^2+^→Pb[OOC-…]_2_+H_2_O

2…-COOH+ Pb(OH)^+^→Pb[OOC-…]_2_+2H_2_O

2…-COOH+ Pb(OH)^+^·H_2_O→Pb[OOC-…]_2_+2H_2_O

## 4. Conclusions

We successfully prepared an adsorbent by catalytic oxidation techniques using HA as a raw material, whose excellent adsorption performance was due to inheriting and adding more reactive functional groups rather than surface structures or porous channels. The maximum adsorption capacity reached 297.72 mg·g^−1^, and the pseudo-second-order model suggests that the adsorption process was dominated by chemical action. The adsorption isotherm indicates multilayer absorption on inhomogeneous surfaces, and the adsorption process was favorable for 1/n was between 0 and 1. Therefore, HA was a promising carbonaceous material for the preparation of adsorbents for wastewater treatment.

## Figures and Tables

**Table 1:** BET surface area and pore sizes of HA, OBC, and HA-400.

Materials	S_BET_/(m^2^·g^−1^)	D_P_/(nm)
HA	2.10	22.43
OBC	16.50	24.81
HA-400	50.25	19.01

**Table 2:** Roman’s D band and G band of HA and OBC.

Sample	*I_D_*	*I_G_*	*I_D_/I_G_*
HA	3028.382	2932.124	1.033
OBC	1347.552	1487.094	0.906

**Table 3:** Comparison of Pb(II) removal from biochar using different adsorbents.

Adsorbent	Adsorption Capacity (mg g^−1^)	References
E-BC	67.07	[11]
bentonite-chitosan (Bt-Ch) composites	94.60 ± 5.63	[41]
AFFA/BC	110.29	[42]
SAMAP	121.43	[43]
HA-400	135.32	This work
HA	232.00	This work
Mango seed (MS)	263.40	[44]
OBC	297.72	This work

**Table 4:** Langmuir, Freundlich adsorption isotherm constants related to Pb(II) onto OBC and correlation coefficients.

	Langmuir			Freundich		
Sample	q_e_ (mg·g^−1^)	K_L_ (L·g^−1^)	R^2^	K_F_ (L·g^−1^)	n_F_	R^2^
OBC	295.86	0.375	0.999	85.88	6.30	0.936

**Table 5:** Dubinin-Radushkevich isotherm model constants related to the adsorption isotherms of Pb(II) onto OBC and correlation coefficients.

Dubinin-Radushkevich	Q_d_ (mg·g^−1^)	β (mol^2^·kJ^−2^)	E_DR_ (kJ·mol^−1^)	R^2^
	251.31	0.037	19.17	0.82764

**Table 6:** Pseudo-first-order kinetic and Pseudo-second-order kinetic parameters for the adsorption of Pb(II) on OBC.

	Pseudo-First-Order Kinetic Model			Pseudo-Second-Order Kinetic Model		
Sample	k_1_ (min^−1^)	q_e_ (mg·g^−1^)	R^2^	k_2_ (min^−1^)	q_e_ (mg·g^−1^)	R^2^
OBC	0.053	275.00	0.9249	1.03×10^−3^	283.29	0.9997

**Table 7:** Thermodynamic parameters for adsorption of mercury.

Parameters	298 K	308 K	325 K	R^2^
ln k_d_	4.24	8.04	9.77	0.99
ΔG (kJ·mol^−1^)	−10.50	−20.59	−25.83	
ΔH (kJ·mol^−1^)	272.52			
ΔS (J·(mol·K)^−1^)	947.80			

**Table 8:** Calculation of adsorption energy.

Complex	E(A…B) (Ha)	E(A) (Ha)	E(B) (Ha)	ΔE (Kcal·mol^−1^)
coronene-OH….Pb(II)	−20,565.85	−1036.32	−19,529.48	−27.10
coronene-COOH….Pb(II)	−20,639.93	−1110.40	−19,529.48	−33.11
coronene-C=O….Pb(II)	−20,564.69	−1035.13	−19,529.48	−26.82
coronene-C-O-C….Pb(II)	−20,605.14	−1075.61	−19,529.48	−26.76

## Data Availability

Data are contained within the article.

## References

[1] Doskočil L., Grasset L., Válková D., Pekař M. (2014). Hydrogen peroxide oxidation of HAs and lignite. Fuel.

[2] Wei Y., Liu H., Liu C., Luo S., Liu Y., Yu X., Ma J., Yin K., Feng H. (2018). Fast and efficient removal of As(III) from water by CuFe_2_O_4_ with peroxymonosulfate: Effects of oxidation and adsorption. Water Res..

[3] Mahar F.K., He L., Wei K., Mehdi M., Zhu M., Gu J., Zhang K., Khatri Z., Kim I. (2019). Rapid adsorption of lead ions using porous carbon nanofibers. Chemosphere.

[4] Sone H., Fugetsu B., Tanaka S. (2009). Selective elimination of lead(II) ions by alginate/polyurethane composite foams. J. Hazard. Mater..

[5] Paz S., Rubio C., Frías I., Gutiérrez J., González-Weller D., Martín V., Revert C., Hardisson A. (2018). Toxic metals (Al, Cd, Pb and Hg) in the most consumed edible seaweeds in Europe. Chemosphere.

[6] Lewis R.D., Condoor S., Batek J., Ong K.H., Backer D., Sterling D., Siria J., Chen J.J., Ashley P. (2005). Removal of Lead Contaminated Dusts from Hard Surfaces. Environ. Sci. Technol..

[7] Li X., Xu W., Yang Y., Li B., Pan G., Chen N., Xie Q. (2023). Optimization of diatom-based blotting materials and their efficient selective adsorption of Pb(II). Mater. Today Commun..

[8] Yang L., Wei J., Liu Z., Wang J., Wang D. (2015). Material prepared from drinking waterworks sludge as adsorbent for ammonium removal from wastewater. Appl. Surf. Sci..

[9] Qu W., Wang H., Li G., Song Z., Liu X., Zhang F., Liu W., Yu D., Ji D. (2023). Efficient Removal of Pb(II), Cr(VI), and Tetracycline Hydrochloride from Aqueous Solutions Using UiO-66-AMP@PAN: Thermodynamics, Kinetics, and Isothermal Adsorption. J. Environ. Chem. Eng..

[10] Khare N., Bajpai J., Bajpai A. (2018). Graphene coated iron oxide (GCIO) nanoparticles as efficient adsorbent for removal of chromium ions: Preparation, characterization and batch adsorption studies. Environ. Nanotechnol. Monit. Manag..

[11] Kim J.-G., Kim H.-B., Baek K. (2023). Novel electrochemical method to activate biochar derived from spent coffee grounds for enhanced adsorption of lead (Pb). Sci. Total. Environ..

[12] Peng W., Li H., Liu Y., Song S. (2016). Comparison of Pb(II) adsorption onto graphene oxide prepared from natural graphites: Diagramming the Pb(II) adsorption sites. Appl. Surf. Sci..

[13] Liu Q., Chen Z., Tang J., Luo J., Huang F., Wang P., Xiao R. (2022). Cd and Pb immobilisation with iron oxide/lignin composite and the bacterial community response in soil. Sci. Total. Environ..

[14] Küçük M.E., Makarava I., Kinnarinen T., Häkkinen A. (2023). Simultaneous adsorption of Cu(II), Zn(II), Cd(II) and Pb(II) from synthetic wastewater using NaP and LTA zeolites prepared from biomass fly ash. Heliyon.

[15] Georgi A., Schierz A., Trommler U., Horwitz C.P., Collins T.J., Kopinke F.-D. (2007). HA modified Fenton reagent for enhancement of the working pH range. Appl. Catal. B Environ..

[16] Cui Z., Xu G., Ormeci B., Hao J. (2023). A novel magnetic sludge biochar was prepared by making full use of internal iron in sludge combining KMnO4-NaOH modification to enhance the adsorption of Pb(II), Cu(II) and Cd(II). Environ. Res..

[17] Yang L., Jin X., Lin Q., Owens G., Chen Z. (2023). Enhanced adsorption and reduction of Pb(II) and Zn(II) from mining wastewater by carbon@nano-zero-valent iron (C@nZVI) derived from biosynthesis. Sep. Purif. Technol..

[18] Gusain R., Kumar N., Fosso-Kankeu E., Ray S.S. (2019). Efficient Removal of Pb(II) and Cd(II) from Industrial Mine Water by a Hierarchical MoS_2_/SH-MWCNT Nanocomposite. ACS Omega.

[19] Kabiri S., Tran D.N., Altalhi T., Losic D. (2014). Outstanding adsorption performance of graphene–carbon nanotube aerogels for continuous oil removal. Carbon.

[20] Dasgupta A., Matos J., Muramatsu H., Ono Y., Gonzalez V., Liu H., Rotella C., Fujisawa K., Cruz-Silva R., Hashimoto Y. (2018). Nanostructured carbon materials for enhanced nitrobenzene adsorption: Physical vs. chemical surface properties. Carbon.

[21] You Y., Jin X., Wen X., Sahajwalla V., Chen V., Bustamante H., Joshi R. (2018). Application of graphene oxide membranes for removal of natural organic matter from water. Carbon.

[22] Yang K., Lou Z., Fu R., Zhou J., Xu J., Baig S.A., Xu X. (2018). Multiwalled carbon nanotubes incorporated with or without amino groups for aqueous Pb(II) removal: Comparison and mechanism study. J. Mol. Liq..

[23] Lipczynska-Kochany E. (2018). Humic substances, their microbial interactions and effects on biological transformations of organic pollutants in water and soil: A review. Chemosphere.

[24] Deng F., Cao Z., Luo Y., Wang R., Shi H., Li D. (2023). Production of artificial HA from corn straw acid hydrolysis residue with biogas slurry impregnation for fertilizer application. J. Environ. Manag..

[25] Li H., Zeng Q., Zhu J., Zhu Y., Xu Y. (2023). Integrated production of humic-like acid, fulvic-like acid, and fermentable sugars from industrial xylooligosaccharides manufacturing waste residues via hydrothermal pretreatment. Ind. Crops Prod..

[26] Shao Y., Luo Z., Bao M., Huo W., Ye R. (2023). Muhammad Ajmal, Wenjing Lu, Enhanced production of hydrothermal HA in a two-step hydrothermal process with acid hydrothermal solution recycling. Chem. Eng. J..

[27] Iftekhar S., Poddar S., Rauhauser M., Daniel, Snow D., David, Hage S. (1239). Preparation of entrapment-based microcolumns for analysis of drug-HA interactions by high-performance affinity chromatography. Anal. Chim. Acta.

[28] Tian K., Zhang J., Zhou C., Liu H., Pei Y., Zhang X., Yan X. (2023). Revealing the roles of carbonized HA in biohydrogen production. Bioresour. Technol..

[29] Yoo M.J., Park H.B. (2018). Effect of hydrogen peroxide on properties of graphene oxide in Hummers method. Carbon.

[30] Baskaran P., Abraham M. (2022). Adsorption of cadmium (Cd) and lead (Pb) using powdered activated carbon derived from Cocos Nucifera waste: A kinetics and equilibrium study for long-term sustainability. Sustain. Energy Technol. Assess..

[31] Yasmeen K., Nawaz S., Iqbal A., Siddiqui A., Umar A.R., Muhammad H., Shafique M., Shah F., Tahir S., Khan A.M. (2022). Removal of Pb(II) from water samples using surface modified core/shell CdZnS/ZnS QDs as adsorbents: Characterization, adsorption, kinetic and thermodynamic studies. Arab. J. Chem..

[32] Wang H., Wang S., Wang S., Tang J., Chen Y., Zhang L. (2021). Adenosine-functionalized UiO-66-NH2 to efficiently remove Pb(II) and Cr(VI) from aqueous solution: Thermodynamics, kinetics and isothermal adsorption. J. Hazard. Mater..

[33] Dong J., Shen L., Shan S., Liu W., Qi Z., Liu C., Gao X. (2021). Optimizing magnetic functionalization conditions for efficient preparation of magnetic biochar and adsorption of Pb(II) from aqueous solution. Sci. Total Environ..

[34] Altynbaeva L.S., Mashentseva A.A., Aimanova N.A., Zheltov D.A., Shlimas D.I., Nurpeisova D.T., Barsbay M., Abuova F.U., Zdorovets M.V. (2023). Eco-Friendly Electroless Template Synthesis of Cu-Based Composite Track-Etched Membranes for Sorption Removal of Lead(II) Ions. Membranes.

[35] Vázquez-Sánchez A.Y., Lima E.C., Abatal M., Tariq R., Santiago A.A., Alfonso I., Aguilar C., Vazquez-Olmos A.R. (2023). Biosorption of Pb(II) Using Natural and Treated *Ardisia compressa* K. Leaves: Simulation Framework Extended through the Application of Artificial Neural Network and Genetic Algorithm. Molecules.

[36] Zhu R., Zhang P., Zhang X., Yang M., Zhao R., Liu W., Li Z. (2021). Fabrication of synergistic sites on an oxygen-rich covalent organic framework for efficient removal of Cd(II) and Pb(II) from water. J. Hazard. Mater..

[37] Lin Z., Weng X., Ma L., Sarkar B., Chen Z. (2019). Mechanistic insights into Pb(II) removal from aqueous so-lution by green reduced graphene oxide. J. Colloid Interface Sci..

[38] Ma Q., Yu Y., Sindoro M., Anthony, Fane G., Wang R., Zhang H. (2017). Carbon-Based Functional Materials Derived from Waste for Water Remediation and Energy Storage. Adv. Mater..

[39] Hong C., Dong Z., Zhang J., Zhu L., Che L., Mao F., Qiu Y. (2022). Effectiveness and mechanism for the simultaneous adsorption of Pb(II), Cd(II) and As(III) by animal-derived biochar/ferrihydrite composite. Chemosphere.

[40] Wang X., Cai D., Ji M., Chen Z., Yao L., Han H. (2022). Isolation of heavy metal-immobilizing and plant growth-promoting bacteria and their potential in reducing Cd and Pb uptake in water spinach. Sci. Total. Environ..

[41] Majiya H., Clegg F., Sammon C. (2023). Bentonite-Chitosan composites or beads for lead (Pb) adsorption: Design, preparation, and characterisation. Appl. Clay Sci..

[42] Yun X., Ma Y., Zheng H., Zhang Y., Cui B., Xing B. (2022). Pb(II) adsorption by biochar from copyrolysis of corn stalks and alkali-fused fly ash. Biochar.

[43] Li L., Bai Y., Qi C., Du Y., Ma X., Li Y., Wu P., Chen S., Zhang S. (2023). Adsorption of Pb(II) and Cu(II) by succinic anhydride-modified apple pomace. Biochem. Eng. J..

[44] Wang Q., Wang Y., Yang Z., Han W., Yuan L., Zhang L., Huang X. (2022). Efficient removal of Pb(II) and Cd(II) from aqueous solutions by mango seed biosorbent. Chem. Eng. J. Adv..

[45] Zhou K., Yang Z., Liu Y., Kong X. (2015). Kinetics and equilibrium studies on biosorption of Pb(II) from aqueous solution by a novel biosorbent: Cyclosorus interruptus. J. Environ. Chem. Eng..

[46] Putranto A., Ng Z.W., Hadibarata T., Aziz M., Yeo J.Y.J., Ismadji S., Sunarso J. (2022). Effects of pyrolysis temperature and impregnation ratio on adsorption kinetics and isotherm of methylene blue on corn cobs activated carbons. S. Afr. J. Chem. Eng..

[47] Majd M.M., Kordzadeh-Kermani V., Ghalandari V., Askari A., Sillanpää M. (2021). Adsorption isotherm models: A comprehensive and systematic review (2010−2020). Sci. Total. Environ..

[48] Sepehri S., Kanani E., Abdoli S., Rajput V.D., Minkina T., Lajayer B.A. (2023). Pb(II) Removal from Aqueous Solutions by Adsorption on Stabilized Zero-Valent Iron Nanoparticles—A Green Approach. Water.

[49] Parmanbek N., Sütekin D.S., Barsbay M., Mashentseva A.A., Zheltov D.A., Aimanova N.A., Jakupova Z.Y., Zdorovets M.V. (2022). Hybrid PET Track-Etched Membranes Grafted by Well-DefinedPoly(2-(dimethylamino)ethylmethacrylate) Brushes and Loadedwith Silver Nanoparticles for theRemoval of As(III). Polymers.

[50] Akpomie K.G., Conradie J. (2021). Isotherm, kinetic, thermodynamics and reusability data on the adsorption of antidepressant onto silver nanoparticle-loaded biowaste. Data Brief.

[51] Mashentseva A.A., Aimanova N.A., Parmanbek N., Temirgaziyev B.S., Barsbay M., Zdorovets M.V. (2022). *Serratula coronata* L. Mediated Synthesis of ZnO Nanoparticles and Their Application for the Removal of Alizarin Yellow R by Photocatalytic Degradation and Adsorption. Nanomaterials.

[52] Weng X., Wu J., Ma L., Owens G., Chen Z. (2018). Impact of synthesis conditions on Pb(II) removal efficiency from aqueous solution by green tea extract reduced graphene oxide. Chem. Eng. J..

[53] Mashentseva A.A., Seitzhapar N., Barsbay M., Aimanova N.A., Alimkhanova A.N., Zheltov D.A., Zhumabayev A.M., Temirgaziev B.S., Sadyrbekov A.A.A.D.T. (2023). Adsorption isotherms and kinetics for Pb(ii) ion removal from aqueous solutions with biogenic metal oxide nanoparticles. RSC Adv..

[54] Suwannahong K., Sirilamduan C., Deepatana A., Kreetachat T., Wongcharee S. (2022). Characterization and Optimization of Polymeric Bispicolamine Chelating Resin: Performance Evaluation via RSM Using Copper in Acid Liquors as a Model Substrate through Ion Exchange Method. Molecules.

[55] Suwannahong K., Wongcharee S., Kreetachart T., Sirilamduan C., Rioyo J., Wongphat A. (2021). Evaluation of the Microsoft Excel Solver Spreadsheet-Based Program for Nonlinear Expressions of Adsorption Isotherm Models onto Magnetic Nanosorbent. Appl. Sci..

[56] Khoso W.A., Haleem N., Baig M.A., Jamal Y. (2021). Synthesis, characterization and heavy metal removal efficiency of nickel ferrite nanoparticles (NFN’s). Sci. Rep..

